# Comparison of air pollution in Shanghai and Lanzhou based on wavelet transform

**DOI:** 10.1007/s11356-017-8959-8

**Published:** 2017-04-21

**Authors:** Yana Su, Yongzhong Sha, Guangyu Zhai, Shengliang Zong, Jiehua Jia

**Affiliations:** 1 School of Management, Lanzhou University, Lanzhou, 730000 China; 2 College of Economics and Management, Lanzhou Institute of Technology, Lanzhou, 730050 China; 3 School of Economics and Management, Lanzhou University of Technology, Lanzhou, 730050 China

**Keywords:** Air pollution index, Wavelet transform, Time series, Change period, Timescale, Shanghai, Lanzhou

## Abstract

For a long-period comparative analysis of air pollution in coastal and inland cities, we analyzed the continuous Morlet wavelet transform on the time series of a 5274-day air pollution index in Shanghai and Lanzhou during 15 years and studied the multi-scale variation characteristic, main cycle, and impact factor of the air pollution time series. The analysis showed that (1) air pollution in the two cities was non-stationary and nonlinear, had multiple timescales, and exhibited the characteristics of high in winter and spring and low in summer and autumn. (2) The monthly variation in air pollution in Shanghai was not significant, whereas the seasonal variation of air pollution in Lanzhou was obvious. (3) Air pollution in Shanghai showed an ascending tendency, whereas that in Lanzhou presented a descending tendency. Overall, air pollution in Lanzhou was higher than that in Shanghai, but the situation has reversed since 2015. (4) The primary cycles of air pollution in these two cities were close, but the secondary cycles were significantly different. The aforementioned differences were mainly due to the impact of topographical and meteorological factors in Lanzhou, the weather process and the surrounding environment in Shanghai. These conclusions have reference significance for Shanghai and Lanzhou to control air pollution. The multi-timescale variation and local features of the wavelet analysis method used in this study can be applied to varied aspects of air pollution analysis. The identification of cycle characteristics and the monitoring, forecasting, and controlling of air pollution can yield valuable reference.

## Introduction

Increasing urbanization in China in the past 20 years has caused many social and environmental problems, such as urban air pollution. Urban air pollution is of increasing global concern because of its enormous impact on the environment, climate, and public health. Similar to most developing countries, China is currently experiencing rapid industrialization and a dramatic increase in energy consumption, both of which have led to air pollution.

Shanghai, the most populous city in the world, is located on the east China coast just to the south of the mouth of the Yangtze River. In the last two decades, Shanghai has been one of the fastest developing cities in the world. The city has become a global financial center and a transport hub with the busiest container port in the world. The expansions of urban population, industrial production, and urban traffic have accelerated the emission of air pollutants. Lanzhou, a typical inland city, is located on the upper reaches of the Yellow River and at the geometric center of the continental territory of China. Lanzhou was once well known for its highest concentrations of gaseous and particulate pollutants (Chu et al. 2008). In recent years, air pollution in Lanzhou has been significantly reduced, and Lanzhou won the “Today Change and Progress Award” in the 2015 Paris Climate Change Conference. By contrast, air pollution in Shanghai has significantly increased. Comparing the air pollution in the two cities can clarify the pollutant characteristics and mechanisms of the temporal evolution of pollution indexes. Such clarification is important to establish reasonable and preventive countermeasures.

Air pollution index (API) is the main index to measure air pollutants; it is influenced by climate, urbanization, manmade pollution sources, and underlying surfaces (Chen et al. 2010; Cheng 2010). The structure of the API system is relatively complex, and the relationship between indicators and results is nonlinear. These features of API time series make the accurate analysis a very difficult task. A feasible method is the wavelet transform, which is a suitable tool for analyzing complex time series and reflecting the different scales of time series and the characteristics of local variation. Wavelet transform in different scales of time series has been commonly applied in many fields, and its usefulness is discussed in other papers (Zaharim et al. 2009; Siwek and Osowski 2012). However, a gap exists between such research fields and API time series research via wavelet transform. In this study, we select Shanghai and Lanzhou as the typical coastal city and inland city respectively. We introduce a one-dimensional continuous wavelet transform (CWT) method to analyze the daily API of the two cities for the past 15 years. Furthermore, we conduct a comparative analysis to reveal the variation regularity of the API time series on multiple timescales.

The structure of this paper is as follows. The “Introduction” section demonstrates the background and motivation of the research. “Standards, data, and methods” section describes air quality standards, data source, and methods. “Results” section shows the results, including the time series characteristics, overall characteristics, change period, and timescale features of API in the two cities. “Discussions” section discusses the reasons for the difference between the API and the main cycle API in the two cities. Finally, “Conclusions” section concludes the research.

## Standards, data, and methods

### Standards and data

The Ambient Air Quality Standards of China (GB 3095-2012), which were issued on February 29, 2012 and implemented since January 1, 2016, were used as evaluation criteria. The Standards stipulate that the limiting values of PM_10_ daily and yearly average concentrations are 150 and 70 μg/m^3^ respectively.

Data were captured from Shanghai and Lanzhou Air Quality Daily, which were provided by the website data center of the Ministry of Environmental Protection of China. The API data started from June 5, 2000 and ended on February 25, 2015. In total, 5274 days were counted, and the serial numbers were from 1 to 5274. The period method was applied to expand the original data to eliminate the boundary effect. The main data process and wavelet transform were executed in MATLAB 7.0.

### Wavelet transform theory

Wavelet is a waveform with amplitude that begins at zero, increases, and then decreases back to zero. Wavelets can be used to extract information from many different kinds of data such as images and audio signals. Wavelet transform is the analysis of time (spatial)–frequency localization, and it can realize multi-scale thinning through dilation and translation of signals (functions). Moreover, this analysis can automatically adapt to the requirement of time–frequency signal analysis and focus on any details of the signal. Signals can be decomposed into the superposition of a series of wavelet functions via wavelet analysis. These wavelet functions are derived from the dilation and translation of a mother wavelet. The irregular wavelet function can approximate the sharp change in non-stationary signals and discrete signals with local feature. Wavelet functions can thus reflect significant real change in the original signal on a timescale. The local analysis characteristic of wavelet transform qualifies it as an effective tool for quantifying non-stationary and discontinuous time series (Stoy et al. 2005). The dilation and translation of a mother wavelet along the time index can generate a series of daughter wavelets, which can be described via scale function (*s*, which is the inverse function of the frequency) and time (*n*) position or translation. A signal can be calculated on different timescales using a series of daughter wavelets, and detailed characteristic scales can be obtained in this process. When the wavelet window is expanded, we can analyze the volatile part of the time series and capture the characteristics of a large-scale (low-frequency) event. When the signal is multiplied by the daughter wavelet, that is, when the signal is uniquely expressed by *s* and *n*, we can calculate the coefficient of the signal in a specific location of the time–frequency domain. If the spectral components in time (*n*) of the signal can be compared with wavelet (*s*), then a relatively large value of wavelet coefficients can be obtained. Accordingly, when we calculate the other combination of *n* and *s*, the signal decomposition in the time–frequency domain can be expressed through a range of coefficients, namely, wavelet transform. We can conclude the fluctuation model of time series through the transform, i.e., cycle dynamic model (Furon et al. 2008; Jevrejeva et al. 2003).

Wavelet transform can be divided into discrete wavelet transform (DWT) and continuous wavelet transform (CWT). DWT is a compact representation of data and commonly applied in engineering and computer science to reduce noise and compress data. CWT is suitable for signal analysis (Grinsted et al. 2004), and widely applied in scientific research to extract the intermittent fluctuation characteristics of time series (Grinsted et al. 2004; Furon et al. 2008).

Unlike Fourier transform, CWT can construct a time–frequency representation of a signal that offers very good time and frequency localization. CWT is also very resistant to the noise in the signal. In CWT, *L*^2^(*R*) and *R* ∈ (−∞, +∞), where *R* is real number and *L*^2^(*R*) is the square-integrable real space on a set of real numbers. If *ψ*(*t*) ∈ *L*^2^(*R*) and meets Eq. (1), then *ψ*(*t*) is named basic wavelet, where *t* is defined as an independent variable in the time domain, that is,

1 $$ c\psi ={\int}_R{\left|{\psi}^{\wedge}\left(\omega \right)\right|}^2/\kern0.5em \left|\omega \right|\kern1em  d\omega \kern1em <\kern1em \infty, $$

where *ω* is an independent variable in the frequency domain (Xu 2002). We can acquire *ψ^*(*ω*) from the Fourier transform of *Ψ*(*t*) and the corresponding continuous wavelet from Eq. (1) as follows:

2 $$ {\psi}_{a, b}(t)={\left| a\right|}^{-1/2}\psi \left( t- b\right)/ a, $$

where *a* , *b* ∈ *R* , *a* > 0. For the arbitrary function *f*(*t*) ∈ *L*^2^(*R*), the CWT of *ψ*_*a* , *b*_(*t*) is

3 $$ {w}_f\left( a, b\right)=\left\{ f(t),{\psi}_{a, b}(t)\right\}{\left| a\right|}^{-1/2}{\int}_R f(t)\psi \left( t- b\right)/ a\kern0.5em  dt, $$

where *a* is a vertical factor (dilation scale), *b* is a horizontal factor (translation scale), and *w*_*f*_(*a*, *b*) is the wavelet coefficient.

The wavelet function for time series analysis should then be selected (Glen and George 2002). Accordingly, Morlet wavelet is selected based on the principles of similarity, discrimination function, and supported length (Wu et al. 2009). This wavelet can clearly identify the random fluctuation and periodicity of time series (Domingues et al. 2005; Andreo et al. 2006), which can be expressed by the following equation, where *C* is constant:

4 $$ \psi (t)={Ce}^{-{t}^2/2} \cos \left(5 t\right). $$

Equation (5) can be used to calculate the main period of the time series and then test the wavelet variance. *W*_*p*_(*a*) is the wavelet variance, which can reflect the intensity of the energy disturbance on different scales in time series, i.e.,

5 $$ {W}_p(a)={\int}_R{\left|{W}_f\left( a, b\right)\right|}^2 db. $$

Wavelet transform assumes that data are circulating. Hence, when dealing with finite-length time series, the edge effect of wavelet power spectrum occurs, i.e., errors occur at the start and end of the power spectrum. On this basis, the total length of the time series must be greater than 2*m* and less than 2*m* + 1 by zero padding at the end of the time series. Nevertheless, when we adopt this measure, the endpoint will not be continuous, or the spectrum amplitude will decrease in the edge of wavelet power spectrum. In this case, the cone of influence (COI) represents the wavelet spectrum region and the corresponding edge effect. On the edge of COI, the value of wavelet spectrum will decline to *e*^−2^ (Torrence and Compo 1998; Grinsted et al. 2004; Furon et al. 2008).

The statistical significance of wavelet power spectrum can be determined according to a null hypothesis, which assumes that the signal is generated from the stable process of a given background power spectrum. The background power spectrum is usually white noise or red noise (Torrence and Compo 1998; Lafrenière and Sharp 2003). Considering that many geophysical time series are of red noise characteristics, i.e., the variance increases as scale increases and frequency decreases, we adopt red noise as the background spectrum to test the wavelet spectrum.

### Implementation steps of wavelet transform

The implementation steps of wavelet transform are as follows:

1. Data preprocessing

The time series data must be consecutive and equal in time steps and standardized.

2. Mother wavelet selection

In this study, the Morlet wavelet is selected as the mother wavelet. For one thing, we aim to obtain smooth and continuous wavelet amplitude in the analysis of time series; thus, the non-orthogonal wavelet function is highly appropriate. For another, we need to select a complex wavelet to obtain the information of amplitude and the phase of time series, because a complex-valued wavelet has an imaginary part that can express the phase well (Torrence and Compo 1998). Furthermore, the Morlet wavelet is a non-orthogonal and exponentially complex-valued wavelet modulated by Gaussian.

3. Scale selection

The time series is 15-year daily mean air pollution data. The time series length is *N* = 5274. We select 5015 data in the interactive wavelet transform to reduce the edge effect of the power spectrum. The time step *dt* = 1, i.e., data for a day. *δ*_*j*_ can be selected as 0.125.

4. Significance test

Given that numerous geophysical time series have red noise characteristics (i.e., variance increases as the scale increases or the frequency decreases), red noise is often used as the background spectrum to test the wavelet spectrum (*lag*1 = 0.72 in the program). In the calculation, values other than COI are estimated at a significance level of 5% at each scale.

### HYSPLIT back-trajectory tool

The gas flow simulation model adopted in “Discussions” section is the Hybrid single-particle Lagrangian-integrated trajectory model (HYSPLIT) from the National Oceanic and Atmospheric Administration (NOAA). The Air Resources Laboratory of the NOAA and the Australian Bureau of Meteorology developed the HYSPLIT-4 model over the past 20 years. This model is a specialized model for calculating and analyzing atmospheric pollutant transport and diffusion track. It features a complete transport, diffusion, and sedimentation model for dealing with the input fields of various meteorological elements, various physical processes, and the functions of different types of pollutant emission sources. The model has been widely used in a variety of pollutants and gases in various areas of transmission and diffusion studies (Cape et al. 2000). In the present study, version 4.9 and the backward transport model are used for track simulation. This model simulates the flow pattern of the target area and is mainly used to interpret the origins of gas or particulate pollutants in the target area. Specifically, we use the HYSPLIT model to trace the source of air pollution in the two cities and then explain the causes of abnormal air pollutant concentrations in the two cities.

## Results

Figure 2 was generated by using Microsoft Excel 2007, while all other figures in this section were generated by MATLAB 7.0.

### Comparison analyses on the time series characteristics of API in the two cities

**Fig. 1:**
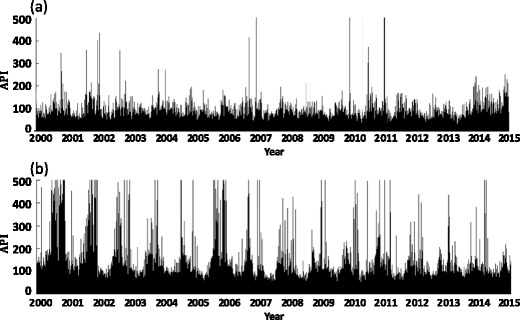
Daily variations of API in Shanghai (**a**) and Lanzhou (**b**) during the last 15 years. The *horizontal axis* started from June 5, 2000 and ended on February 25, 2015; the *vertical axis* represents the daily average value of API, with a maximum value of 500

The change of daily API in Shanghai and Lanzhou over 15 years is shown in Fig. 1. Overall, the air pollution in Lanzhou was higher than that in Shanghai.

Figure 1a shows the number of days when API over 100 was approximately 666 in Shanghai, of which 81% occurred from November to the next April. The maximum value of API was 500 and appeared on April 2, 2007, March 21, 2010, May 2, 2011, and May 3, 2011. Weather records indicated that these days were accompanied by sand and dust weather.

Figure 1b correspondingly presents the number of days when API over 100 was approximately 2103 in Lanzhou, of which 70% occurred from November to the next April. API over 500 was recorded in 57 days, 14 days from November to the next January and 43 days from March to April.

**Fig. 2:**
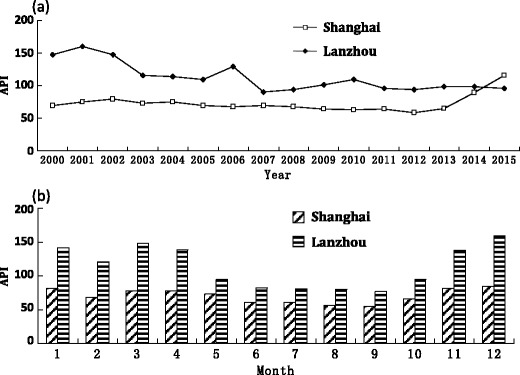
Comparison between the yearly and monthly average values of API in Shanghai (**a**) and Lanzhou (**b**) during the last 15 years. **a** The yearly average value of API, calculated by dividing the sum of daily average API each year and the corresponding days in the year. **b** The monthly average value of API, calculated by dividing the sum of daily average API each month and the corresponding days in the month

Figure 2a presents the comparison diagram of the annual mean of API. The annual average value of API in Shanghai changed between 60 and 70 from 2000 to 2013. The API from 2014 to 2015 increased to 89 and 115, respectively. The API in Shanghai showed an upward trend in general. On the contrary, the annual API average value of Lanzhou decreased from 150 to 100 during 3 years (2000 to 2003). The annual average of API was approximately 110 from 2003 to 2005, peaking at 129 in 2006. From 2007 to 2015, the value fell to 95. A downward trend was generally observed. However, the pollution in Shanghai has become higher than that in Lanzhou since 2015.

The comparison of the monthly mean of API is illustrated in Fig. 2b. The degree of air pollution in Shanghai was not distinctly changeable, whereas the fluctuations in air pollution in Lanzhou were seasonal. The general characteristics in the two cities accorded with the high in winter and spring and low in summer and autumn, and the air pollution deterioration began in November to the next April. The API in February significantly decreased due to the reduced production activities during the Spring Festival period (Most of the Spring Festival is in February of the Gregorian calendar).

### Comparison analyses on the overall characteristics of API in the two cities

**Fig. 3:**
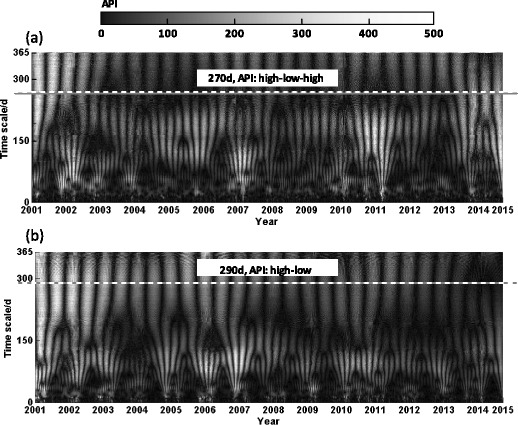
Timescale distribution of API in Shanghai (**a**) and Lanzhou (**b**) during the last 15 years. This figure reflects the API on the corresponding date of different timescales. The pollution variation period is in different timescales, and we can clearly observe the overall pollution variation trend on a larger time scale. The lighter color represents the higher API and the lower API conversely

Figure 3 shows the cyclic variation of API in the two cities during 5274 days on different timescales (the horizontal axis is the year, and the vertical axis is the timescale on a daily basis). The value of the wavelet coefficient can indicate signal intensity changes. Grayscale represents the wavelet coefficient in the figure; high grayscale corresponds to small wavelet coefficient and light air pollution, and vice versa. For a given timescale, we can analyze the characteristics of the two-dimensional distribution of API on the timescale according to the wavelet coefficients displayed by signal change. Different frequencies of API are on different scales, i.e., high frequency on small scale and low frequency on large scale (Wang et al. 2011). The general characteristics of API can be clearly observed on a larger scale.

Figure 3a suggests that the air pollution in Shanghai on the larger scale (270 days) presents a high–low–high phenomenon. The values of API were high in 2001 and 2002 and then decreased. Nevertheless, the API has increased over the past 4 years.

Figure 3b shows that the degree of pollution in Lanzhou presents a more obvious descending trend on the larger scale (290 days) and periodic variation on the medium scale (100 days). Grayscales in winter were relatively low, which means the air pollution was high.

### Comparison analyses on the change period of API in the two cities

Figure 3 reveals that the API changes present both periodicity and feature of large cycles nested in small cycles in the two cities. Wavelet variance analysis can be used to calculate the change period of air pollution and obtain the relative intensity of energy disturbance in time series on different scales (Percival and Walden 2000). The wavelet variance diagram can reflect the main timescale of the time series, which is the main cycle (Chen et al. 2007).

**Fig. 4:**
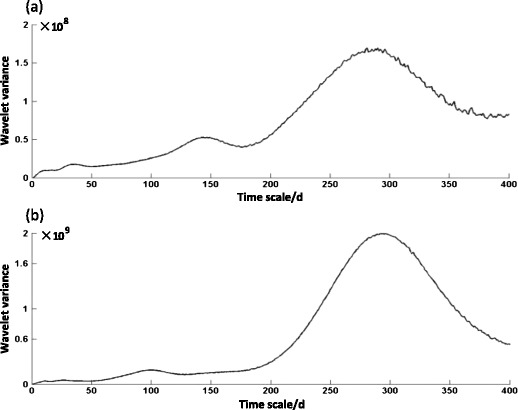
Wavelet variance of API in Shanghai (**a**) and Lanzhou (**b**) during the last 15 years. The *horizontal axis* reflects the timescale, with a maximum of 400 days; the *vertical axis* shows the wavelet variance, which represents the scale fluctuation in the time series, and the scale of its peak is called the main timescale, which can reflect the main period of the time series

Figure 4a shows that the curve has three peaks, namely, 270-day main cycle, 150-day sub-cycle, and 40-day small cycle of API in Shanghai in the past 15 years. Although other peaks occurred in the position, after the white noise test, they were removed as noise.

Figure 4b displays that the 290-day main cycle and 100-day sub-cycle of API occurred in Lanzhou in the past 15 years. Although a peak existed under 50 days, it was removed as noise because it was not significant in the white noise test.

### Comparison analyses on the timescale features of API in the two cities

We analyze the main cycle and sub-cycle of air pollution to compare the characteristics of API time series change on large, medium, and small scales in the two cities. The quasi-one year (365 days), the main period (270 days), and the sub-period (150 days) are taken as the long, medium, and short timescales of Shanghai, respectively; and the quasi-one year (365 days), the main period (290 days), and the sub-period (100 days) as the long, medium, and short timescales of Lanzhou, respectively, to analyze the characteristics of the API time series change of Shanghai and Lanzhou.

**Fig. 5:**
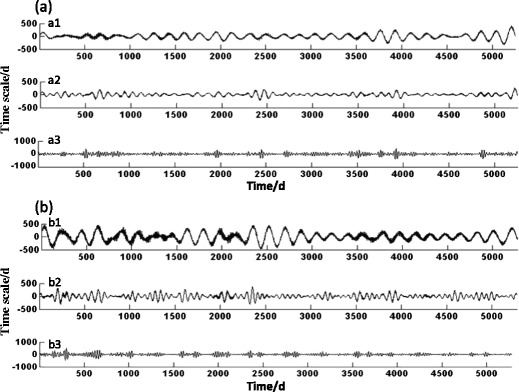
Wavelet coefficients of API during the last 15 years at given scales. The wavelet coefficients of API in Shanghai on timescales of 365, 270, and 150 days are given in *a1*, *a2*, and *a3*, respectively, and the wavelet coefficients of API in Lanzhou on timescales of 365, 290, and 100 days are provided in *b1*, *b2*, and *b3*, respectively

Figure 5 exhibits the air pollution changes in the two cities on various timescales. Figure 5(a1) shows the low–high periodic oscillation at the main cycle (270 days) of API in Shanghai. Larger amplitudes in 2002 and 2003 and high pollution in winter were recorded. Other years had relatively small amplitudes and peak differences. The peak in 2007 was slightly higher, and the air pollution was higher in winter. Figure 5(a2 and a3) reflect that the wavelet coefficient curves had similar laws at the sub-cycle (150 days) and small cycle (40 days) respectively, i.e., stronger volatility, shorter oscillation cycle, and higher frequency. However, the former wavelet coefficient curves had larger amplitude in winter in 2002 and 2007 and flat oscillation in other years. The API in 2001–2003 had large amplitude and flat oscillation in the following 3 years. The amplitude in 2007 increased, but the oscillation appeared flat again in the next 2 years. In 2010 and 2015, the amplitude began to increase and the pollution aggravated conversely.

Figure 5(b1) reflects the low–high periodic oscillation at the main cycle (290 days) of API in Lanzhou. A descending tendency generally appeared. Figure 5(b2) shows that the fluctuation in the wavelet coefficients was not very sharp, the oscillation period was short, and the frequency was high in the sub-period scale of 100 days. In the beginning of a few years of winter, the spring amplitude was larger and then became gentle after 2007. Figure 5(b3) shows that the regularity of air pollution in Lanzhou was not distinct on the scale of 50 days.

The main cycle, sub-cycle, and small cycle of API in Shanghai were 9, 5, and 1 month, respectively; those in Lanzhou were 10, 3, and 1.5 months. Wave crest always appeared in winter, whereas trough occurred in summer. In the two cities, the API in the winter was higher, while the degree of air pollution in the summer was relatively better. After the increase in the degree of pollution in winter, a sub peak would appear in the next spring. The preceding results also showed that air pollution in Shanghai has increased in recent years, especially in the spring. By contrast, air pollution in Lanzhou has gradually reduced in general.

## Discussions

### Difference in the APIs of the two cities

The preceding analysis indicates a significant difference between the API in Shanghai and in Lanzhou in the last 15 years. This phenomenon is attributed to three reasons.

**Fig. 6:**
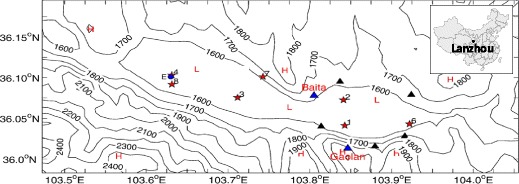
Geography and isobaths of Lanzhou (unit: m). Source: Chu et al. (2008). The figure shows that Lanzhou locates at a narrow (2–8 km width), long (40 km) valley basin (elevation 1500 to 1600 m), with Baita Mountain (above 1700 m elevation) in the north and Gaolan Mountain (above 1900 m elevation) in the south. The topographic characteristics make Lanzhou vulnerable to particulate pollution.

First, the difference in the underlying surface of the two cities leads to the discrepancy in API. Shanghai is located in the east coast of China. The middle-lower Yangtze Plain, where Shanghai is situated in, is low and flat. Except for a few hills lying in the southwest corner, most parts of the Shanghai area are flat. The average sea level elevation is approximately 4 m. Conversely, Lanzhou is located at a narrow (2–8 km width) and long (40 km) valley basin (elevation 1500–1600 m), with the Tibetan Plateau in the west, Baita Mountain (above 1700 m elevation) in the north, and Gaolan Mountain (above 1900 m elevation) in the south (Fig. 6). The aspect ratio of the valley (depth versus width) is approximately 0.07, which blocks the air streams and makes the pollutants difficult to disperse (Chu et al. 2008).

**Fig. 7:**
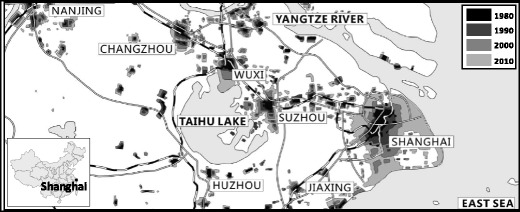
Historic expansion of the Yangtze River delta urban agglomeration in 1980, 1990, 2000, and 2010. Source: Zhu and Zheng (2012). Shanghai is now one of the largest metropolitan areas in the world. Urbanization has led to industrialization and caused severe air pollution

Second, the difference in the surrounding environment of the two cities affects the API considerably. We determine a striking difference with respect to the surrounding environment of the two cities. Specifically, a high degree of urbanization and the developed industry are the real reflection around Shanghai. Thus, the pollution diffusion and stack aggravate the degree of air pollution in Shanghai. Historic expansion of the Yangtze River delta urban agglomeration in 1980, 1990, 2000, and 2010 is shown in Fig. 7 (Zhu and Zheng 2012). Collaborative governance is thus urgently needed to curb the increasingly difficult problem. By contrast, around Lanzhou, the terrain is mainly hills and mountains, and people there depend significantly on agriculture for their livelihood. Therefore, the surrounding environment of Lanzhou has a minimal influence on the air quality, which needs relatively simple governance on air pollution.

**Fig. 8:**
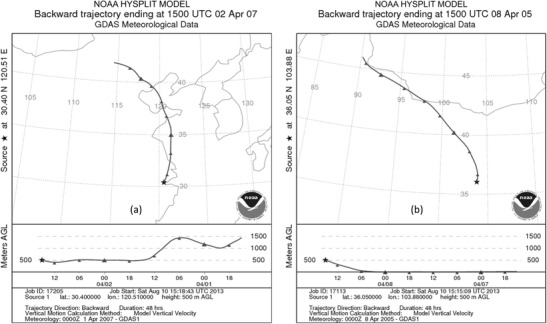
Back-trajectory analysis. **a** Back-trajectories on April 2, 2007 in Shanghai and **b** back-trajectories on April 8, 2005 in Lanzhou. In Fig. 6a, the air current dropped gradually from 1500 to 500 m on April 1, 2007, which brought the dust and sand from Hunshadake Sandy Land, through Bohai Sea, Shandong Peninsula, and Huanghai Sea, and finally delivered to Shanghai. The air current in Fig. 6b is low before six o’clock of April 8, 2005, and then jumped to 500 m after 12 o’clock. This phenomenon brought the dust and sand from Tengger Desert, through Hexi Corridor, and delivered to Lanzhou

Third, the meteorological conditions of the two cities contribute to the difference in API. In winter and spring, the wind blows from the inland to Shanghai. The inland wind transports the pollutants through long distances, and the amount of precipitation decreases, which is affected by climate. Air pollution in Shanghai becomes higher in winter and spring and lower in summer and autumn (Dong et al. 2009). In the spring, dust weather appears in the Hexi Corridor of Gansu Province and leads to a significant increase in total suspended particle concentration in Lanzhou (Tao et al. 2007). Coal use in the winter also increases the degree of air pollution. Figure 8 applies the HYSPLIT to make a back-trajectory analysis (Dou et al. 2012; Li et al. 2010; Qin et al. 2010), which shows the track analysis of back-step 48 h in Shanghai on April 2, 2007 and back-step 24 h in Lanzhou on April 8, 2005. The analysis implies that the severe pollution in the two cities is related to the dust weather. The API of Shanghai on April 2, 2007 was 500, and the growth rate was 331% compared with 48 h prior; the API of Lanzhou on April 8, 2005 was 500, and the growth rate was 338% compared with 24 h prior. The dust in Shanghai was transported by the Hunshadake Sandy Land, whereas air pollution in Lanzhou was transported by the Tengger Desert. The finding is in accordance with that obtained by Wang et al. (2003).

### Main cycle of API in the two cities

The preceding research shows that the main cycle of API in the two cities (270 days in Shanghai and 290 days in Lanzhou) is considerably shorter than a year, which can be attributed to the Blue Sky Project carried out in the two cities to control air pollution. The project decreases the API in winter; in spring and summer, increased dust causes high API accordingly. Nevertheless, whether the air pollution is higher in summer and lower in winter according to the occasional phenomenon is too early for us to conclude. We should omit such special circumstances, such as in 2010, in Fig. 3. A significant difference also exists between the two cities in terms of the sub-period; Shanghai has a sub-period of 150 days, whereas Lanzhou has 100 days. This difference demonstrates a time period of air pollution similar to climate change circle. API peaks occur in the beginning and end of the year in the two cities, whereas API troughs appear in summer.

## Conclusions

Air pollution in the cities of Shanghai and Lanzhou generally share the common features of instability, nonlinearity, and multiple timescales, i.e., higher in winter and spring, lighter in summer and autumn, and had been increasing since November until April of the following year. The pollution days and degree in Lanzhou had been more and higher than those in Shanghai for the past 15 years. The degree change in Shanghai was not significant, whereas the seasonal variation in Lanzhou was obvious.

In the aspect of change trend, the air pollution degree in Shanghai has been rising on the whole. Such a trend has particularly been evident since the beginning of 2013. The pollution degree in Shanghai has been beyond that of Lanzhou since the second half of 2014, which is relevant to the pollution diffusion and stack of the surrounding environment of Shanghai. Conversely, the pollution degree in Lanzhou has decreased generally. The API of Lanzhou has dropped to approximately 95 since 2007. Lanzhou has been removed from the ranks of the top 10 heavily polluted cities of China since 2012 because of the systematic measures of pollution control in Lanzhou.

In another aspect of change period, the main cycle of air pollution in the two cities was similar (Shanghai was 270 days and Lanzhou was 290 days). However, a distinct difference existed in their sub-cycle (Shanghai was 150 days and Lanzhou was 100 days). A small cycle of 40 days appeared in Shanghai, but it was not apparent in Lanzhou. In 1 year, a peak of air pollution occurred every 5 months in Shanghai but appeared every 3 months in the winter and spring in Lanzhou. Such differences were mainly due to the impact of the weather process in Shanghai and the topographical and meteorological factors in Lanzhou.

These conclusions are of great instructive significance for air pollution control in Shanghai and Lanzhou. Wavelet analysis of the multi-timescale variation and local feature method can be applied to varied aspects of air pollution analysis, which have important reference values for identifying cycle characteristics, as well as monitoring, forecasting, and controlling air pollution.

## References

[CR1] Andreo B, Jiménez P, Durán JJ (2006). Climatic and hydrological variations during the last 117-166 years in the south of the Iberian peninsula from spectral and correlation analyses and wavelet continuous analyses. J Hydrol.

[CR2] Cape JN, Methven J, Hudson LE (2000). The use of trajectory cluster analysis to interpret trace gas measurements at Mace Head, Ireland. Atmos Environ.

[CR3] Chen KL, Li SC, Zhou QF (2007). Multi-scale study on climate change for recent 50 years in Dari County in the source regions of the Yangtze and Yellow rivers. Geogr Res.

[CR4] Chen LH, Yu Y, Chen JB (2010). Characteristics of main air pollution in Lanzhou during 2001-2007. Plateau Meteorology.

[CR5] Cheng T (2010). Dependence of ambition air quality on meteorology basing on wavelet method in shanghai.

[CR6] Chu PC, Chen YH, Lu SH (2008). Particulate air pollution in Lanzhou China. Environ Int.

[CR7] Domingues M, Mendes O, Costa AMD (2005). On wavelet techniques in atmospheric sciences. Adv Space Res.

[CR8] Dong JY, Wang SG, Shang KZ (2009). Influence of precipitation on air quality in several cities of China. Journal of Arid Land Resources and Environment.

[CR9] Dou XY, Xu J, Han DH (2012). Transportation characteristics of PM_10_ in the typical pollution weather in Xining. Journal of Meteorology and Environment.

[CR10] Furon AC, Wagner-Riddle C, Smith CR et al (2008) Wavelet analysis of wintertime and spring thaw CO_2_, and N_2_O fluxes from agricultural fields. Agric For Meteorol 148(8):1305–1317

[CR11] Glen SW, George WB (2002). Wavelet-based correlation (WBC) of zoned crystal populations and magma mixing. Earth & Planetary Science Letters.

[CR12] Grinsted A, Moore JC, Jevrejeva S (2004). Application of the cross wavelet transform and wavelet coherence to geophysical time series. Nonlinear Process Geophys.

[CR13] Jevrejeva S, Moore JC, Grinsted A (2003). Influence of the Arctic Oscillation and El Niño-Southern Oscillation (ENSO) on ice conditions in the Baltic Sea: the wavelet approach. Journal of Geophysical Research: Atmospheres.

[CR14] Lafrenière M, Sharp M (2003). Wavelet analysis of inter-annual variability in the runoff regimes of glacial and nival stream catchments, Bow lake, Alberta. Hydrol Process.

[CR15] Li DP, Cheng XH, Yu YT (2010). Effects of meteorological factors on air quality above the third grade pollution in Beijing. Journal of Meteorology and Environment.

[CR16] Percival DB, Walden AT (2000). Wavelet methods for time series analysis.

[CR17] Qin YY, Wang J, Cheng JG (2010). A typical air pollution event caused by external source transport during 2008 in Qingdao, Shandong province. Journal of Meteorology and Environment.

[CR18] Siwek K, Osowski S (2012). Improving the accuracy of prediction of pm_10_ pollution by the wavelet transformation and an ensemble of neural predictors. Eng Appl Artif Intell.

[CR19] Stoy PC, Katul GG, Siqueira MB (2005). Variability in net ecosystem exchange from hourly to inter-annual time scales at adjacent pine and hardwood forests: a wavelet analysis. Tree Physiol.

[CR20] Tao JH, Huang YX, Lu DR (2007). Influence of sand-dust activities in Hexi corridor on PM_10_ concentration in Lanzhou and its assessment. J Desert Res.

[CR21] Torrence C, Compo GP (1998). A practical guide to wavelet analysis. Bull Am Meteorol Soc.

[CR22] Wang SG, Wang JY, Zhou ZJ (2003). Regional characteristics of dust events in China. J Geogr Sci.

[CR23] Wang HP, Zhang B, Liu ZH (2011). Wavelet analysis of air pollution index changes in Lanzhou during the last decade. Acta Sci Circumst.

[CR24] Wu XL, Zhang B, Ai NS (2009). Wavelet analysis on SO_2_ pollution index changes of shanghai in recent 10 years. Environmental Science.

[CR25] Xu JH (2002). Mathematical methods in contemporary geography.

[CR26] Zaharim A, Shaharuddin M, Karim OA (2009). Relationships between airborne particulate matter and meteorological variables using non-decimated wavelet transform. Eur J Sci Res.

[CR27] Zhu Z, Zheng B (2012). Study on spatial structure of Yangtze river delta urban agglomeration and its effects on urban and rural regions. Journal of Urban Planning & Development.

